# Health Issues in Postmenopausal Women Who Drink

**Published:** 2002

**Authors:** Thomas C. Register, J. Mark Cline, Carol A. Shively

**Affiliations:** Thomas C. Register, Ph.D., and J. Mark Cline, D.V.M., Ph.D., are associate professors, and Carol A. Shively, Ph.D., is a professor, all in the Department of Pathology, Section on Comparative Medicine, Wake Forest University School of Medicine, Winston-Salem, North Carolina

**Keywords:** female, postmenopause, AOD (alcohol and other drug) use, alcoholic beverage, beneficial vs adverse drug effect, risk factors, estrogens, heart disorder, osteoporosis, breast, cancer *

## Abstract

Alcohol use may impact the health and well-being of postmenopausal women through the direct interaction of alcohol with organ systems when alcohol is ingested, transported through the blood, and processed for removal, and by indirect means through the modification of blood concentrations of sex hormones. Circulating hormones are known to affect risk for coronary heart disease, osteoporosis, and breast cancer, which are major causes of significant illness and death in postmenopausal women. The effects of alcohol consumption on these three diseases in postmenopausal women are reviewed, along with limitations in interpreting results of studies of alcohol’s effects on postmenopausal women’s health.

The cessation of ovarian function at menopause and the accompanying decline in the production of steroid hormones secreted by the ovaries (i.e., sex steroids) create a unique set of health concerns for women. Declines in sex steroid levels—particularly estrogen—have been associated with a variety of diseases and conditions, including coronary heart disease (CHD), osteoporosis, cognitive dysfunction, urinary incontinence, hot flushes, and mood changes, among others.

Alcohol use affects the health of post-menopausal women directly by its direct impact on organ systems such as the brain, liver, and gastrointestinal system,^1^ and may also affect health indirectly by altering blood concentrations of sex steroids that affect the risk for disease. Endogenous and exogenous sex steroids^2^ are known to influence the risk for diseases that are major causes of significant illness and death in postmenopausal women. In particular, estrogen has beneficial effects on postmenopausal women’s bone health and has long been associated with protection against CHD in women (Nelson et al. 2002). Estrogen may, however, increase women’s breast cancer risk (Grady et al. 1992). This article reviews research on the effects of alcohol consumption on CHD, osteoporosis, and breast cancer in postmenopausal women and notes limitations to interpreting current epidemiological data on alcohol’s potential effects on post-menopausal health.

## Alcohol and Estrogens

Several studies suggest that, in pre- and postmenopausal women, light-to-moderate alcohol consumption may increase blood concentrations of estrogen and the byproducts of estrogen metabolism (Dorgan et al. 2001; Gavaler et al. 1991; Gavaler and Van Thiel 1992; Mendelson and Mello 1988; Mendelson et al. 1988; but see Longnecker 1999, and Purohit 1998). (See table 1 for definitions of light, moderate, and heavy drinking in women.) For example, a recent study of postmenopausal women of several nationalities demonstrated a positive association between moderate alcohol consumption and circulating estrogen levels in all groups except African American women, for whom the sample size was small (Gavaler et al. 2002).

The major source of naturally occurring estrogens in postmenopausal women is the enzymatic conversion of sex hormones known as androgens to estrogen, a process called aromatization. Alcohol consumption increases aromatization and may reduce the rate at which one type of endogenous estrogen, estradiol, is cleared from the blood (Gavaler and Van Thiel 1992; Ginsburg 1999; Gordon et al. 1975; Wimalasena et al. 1993).

Thus, alcohol use may exert its effects on the risk of CHD, osteoporosis, and breast cancer in part through increasing the production of endogenous estrogens (Tivis and Gavaler 1994). For most women, alcohol consumption is likely to have begun before menopause and to have simply continued into postmenopausal years, rather than beginning suddenly after menopause. As noted above, alcohol has effects on health that result from direct exposure to alcohol during ingestion (specifically affecting the gastrointestinal tract and the liver) and while in the bloodstream, and has another set of effects related to the modification of blood hormones. However, it is difficult to precisely define the relative contributions of these because of the complexity of alcohol’s effects.

## Coronary Heart Disease

Women have a low incidence of CHD until after menopause, apparently because estrogen protects against the disease (Colditz et al. 1987). After menopause, women’s estrogen levels decline sharply, and their risk of CHD gradually increases, approaching the risk observed in men. In both men and women, CHD is the leading cause of death. Animal studies indicate that administering estrogens (including the most common form of the hormone used in hormone replacement therapy, conjugated equine estrogens) to healthy female subjects at the onset of estrogen deficiency protects against coronary artery disease (Adams et al. 1990, 1997).^3^

**Figure 1:**
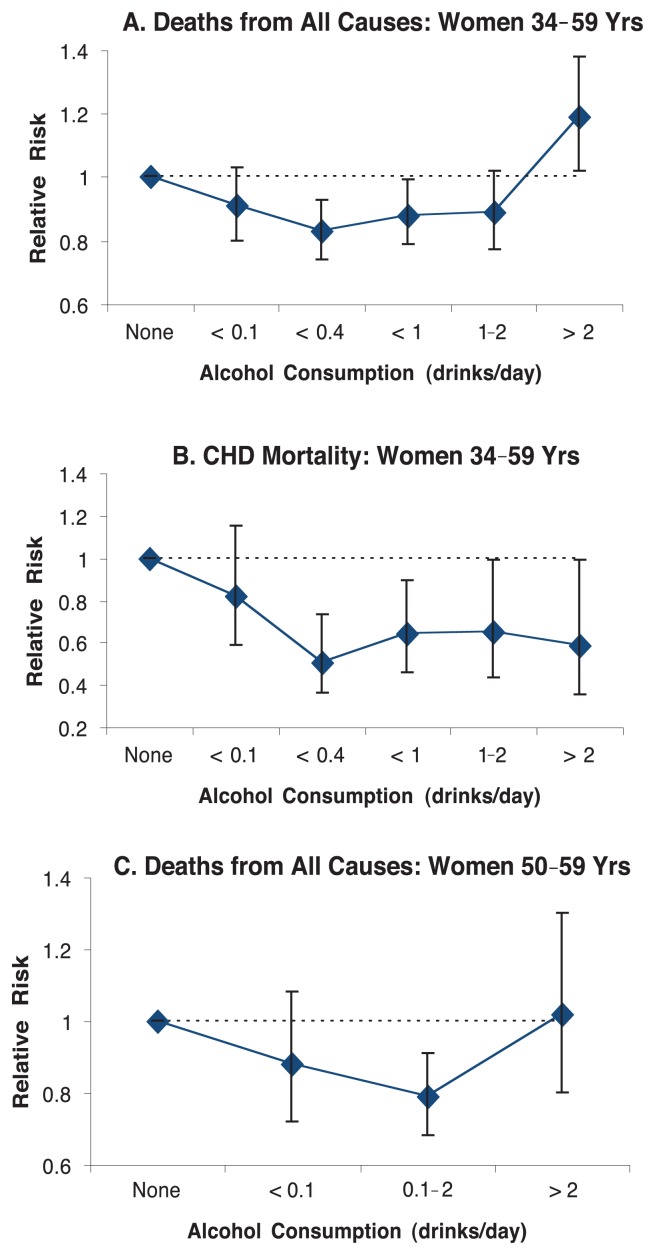
Data from the Nurses’ Health Study, which examined risk factors for chronic disease in a group of 85,709 women ages 34 to 59, showed that both (A) rates of death from all causes and (B) rates of death from coronary heart disease (CHD) were reduced for women who consumed one drink per week (light drinkers) and remained low for those who consumed up to two drinks per day or more (moderate-to-heavy drinkers). This benefit was greater among women who had at least one CHD risk factor (about 73 percent of subjects), and it increased as women grew older. Even among women over age 50 (i.e., primarily postmenopausal women), the relative risk of death was similar to the risk observed in the entire group (C). SOURCE: Women’s Health Study (Fuchs et al. 1995).

Many epidemiological studies suggest that moderate alcohol consumption may protect against CHD in both genders (Albert et al. 1999; Camargo et al. 1997; Fuchs et al. 1995; Gaziano et al. 2000). Data from the Nurses’ Health Study (Fuchs et al. 1995), which examined risk factors for chronic disease in a group of 85,709 women ages 34 to 59, showed that total mortality rates (see figure 1A) and rates of death from CHD (see figure 1B) were reduced for women who consumed one drink per week (light drinkers) and remained low for those who consumed up to two drinks per day or more (moderate-to-heavy drinkers).^4^ This benefit was greater among women who had at least one CHD risk factor (about 73 percent of subjects), and it increased as women grew older. Even among women over age 50, the relative risk of death^5^ was similar to the risk observed in the entire group (see figure 1C), suggesting that postmenopausal women are similarly affected. The decreased risk of death in the group of women who consumed between 0.1 and 2 drinks per day (light-to-moderate drinkers) can be attributed primarily to decreases in CHD. At greater than two drinks per day, total mortality increased. Thus, a therapeutic window for alcohol consumption and total mortality may be relatively narrow and at a moderate level (two or fewer drinks per day).

Recent randomized trials in post-menopausal women suggest that mechanisms underlying the protective effect of alcohol on CHD risk may include improvements in risk factors and conditions associated with CHD, such as blood cholesterol levels (i.e., reductions in LDL, or “bad” cholesterol, and increases in HDL, or “good” cholesterol) (Baer et al. 2002) as well as reductions in fasting insulin levels and increased insulin sensitivity (Davies et al. 2002). The critical observation in this research is that relative risks for developing CHD and for CHD death were greater for women who are non-drinkers than for light and moderate drinkers. This finding could lead to the conclusion that—at least from the perspective of cardiac health and where drinking is not contraindicated, as it is in pregnant women—nondrinking women might benefit by becoming moderate drinkers. However, the characteristics of control groups in this area of research need to be considered when drawing conclusions from these data, an issue that is addressed in the section “Interim Summary and Further Considerations.”

**Alcohol and Coronary Heart Disease in Men**

The relationship between alcohol consumption and CHD mortality observed in postmenopausal women appears to be mirrored in men, for whom there is a great deal more information (Camargo et al. 1997; Albert et al. 1999; Gaziano et al. 2000; Mukamal et al. 2003). Data from the Physicians’ Health Study (Camargo et al. 1997; Albert et al. 1999; Gaziano et al. 2000), a prospective cohort study of 22,000 men ages 40 to 84, suggest a marked dose-dependent response to alcohol similar to that observed for women (compare figure 1A in the main article and this sidebar’s figure, below). For death from all causes, the lowest risk of mortality covered a broad range of alcohol consumption, from one drink per week to one drink per day (light-to-moderate drinking), and risk increased as alcohol consumption rose beyond the moderate level (see graph A, below). The relationship between alcohol consumption and sudden death related to coronary heart disease (SCD; symptom duration less than 1 hour) seems to follow a similar pattern (see graph B). On the other hand, the risk for myocardial infarction (MI), or heart attack (see graph C), as well as for nonsudden CHD-related death (graph D), continued to decrease as alcohol consumption increased.

— Thomas C. Register, J. Mark Cline, and Carol A. Shively

**Figure:**
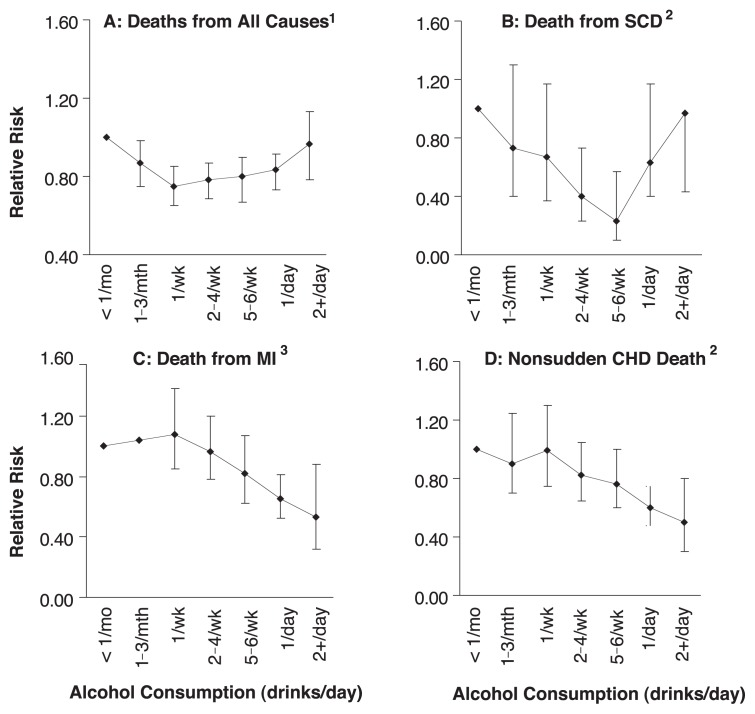
SOURCES: ^1^Gaziano et al. 2000. ^2^Albert et al. 1999. ^3^Camargo et al. 1997.

ReferencesAlbertCMansonJCookNModerate alcohol consumption and the risk of sudden cardiac death among U.S. male physiciansCirculation10094495019991046852510.1161/01.cir.100.9.944CamargoCAJrStampferMJGlynnRJModerate alcohol consumption and risk for angina pectoris or myocardial infarction in U.S. male physiciansAnnals of Internal Medicine12653723751997905428110.7326/0003-4819-126-5-199703010-00005GazianoJGazianoTGlynnRLight-to-moderate alcohol consumption and mortality in the Physicians’ Health Study enrollment cohortJournal of the American College of Cardiology359610520001063626610.1016/s0735-1097(99)00531-8MukamalKJConigraveKMMittlemanMARoles of drinking pattern and type of alcohol consumed in coronary heart disease in menNew England Journal of Medicine34810911820031251992110.1056/NEJMoa022095

## Osteoporosis

Whereas CHD is the leading cause of death in women, osteoporosis-related injuries are a leading cause of disability in postmenopausal women (Cummings and Melton 2002). In the United States, a common late-life scenario in women is the need for residential care because of reduced mobility and chronic pain from vertebral or hip fractures. Osteoporosis is also a major cause of mortality, as hip fractures often lead to complications ending in death. In fact, the future likelihood of a 50-year-old woman dying from hip fracture complications is the same as her likelihood of someday dying from breast cancer (Cummings et al. 1989).

In the general population, alcoholics have reduced bone mineral density (BMD), and male alcoholics have been shown to have an increased risk of fractures (Turner 2000). Research suggests, however, that moderate drinking has beneficial effects on bone, at least in women. (For more information on how chronic heavy drinking affects bone health in older women, see the article in this issue by Sampson.)

**Figure 2:**
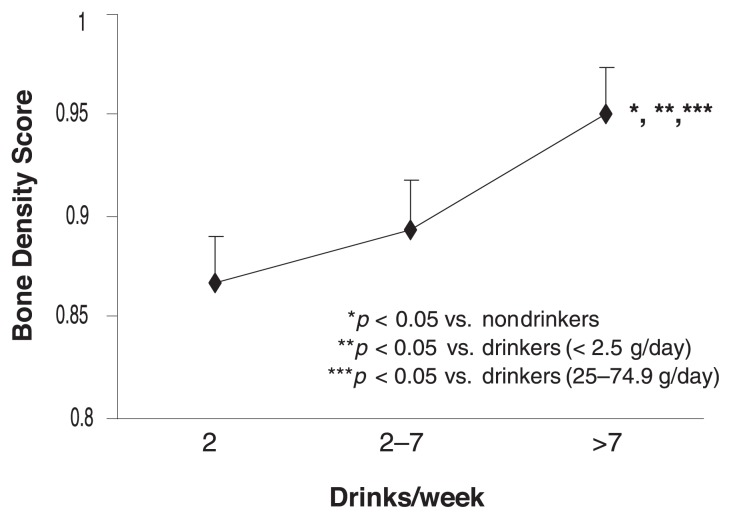
Bone density was studied in 188 white postmenopausal women participating in the Nurses’ Health Study. Women who drank moderately (more than 75 g of alcohol, or about seven drinks, per week) had greater bone density than did nondrinkers or either of the other two groups of drinkers (who consumed fewer than two drinks or between two and seven drinks per week). SOURCE: Feskanich et al. (1999).

Although longitudinal studies of the effects of women’s alcohol consumption on bone are limited, those that have been performed suggest an overall beneficial effect of light-to-moderate alcohol consumption on bone mineral density. Feskanich and colleagues (1999) studied bone density in 188 white postmenopausal women participating in the Nurses’ Health Study (see figure 2). Women who drank moderately (more than 75 grams of alcohol, or about seven drinks, per week) had greater bone density than did non-drinkers or either of the other two groups of drinkers (who consumed fewer than two drinks or between two and seven drinks per week).

A large investigation that followed 13,917 women suggested that moderate alcohol consumption did not increase women’s risk of vertebral or forearm fracture (Hoidrup et al. 1999). Further, a case control study in Sweden suggested a modest decrease in risk of hip fracture among women who stated that they drank alcohol, even at levels ranging from less than 3 grams per day to more than 6 grams per day (Baron et al. 2001). These data suggest that moderate alcohol consumption may have modest beneficial effects on bone for postmenopausal women. Several observational studies suggest a positive effect of moderate alcohol consumption on bone density in older women (Rapuri et al. 2000; Felson et al. 1995).

Nevertheless, the potential skeletal benefits of moderate alcohol consumption in postmenopausal women are relatively minor compared with the benefits of hormone replacement therapy (HRT) (Nelson et al. 2002) or other treatments such as biphosphonates (Levis et al. 2002). For example, in the Postmenopausal Estrogen/Progestin Interventions (PEPI) trial of 875 post-menopausal women, alcohol use was associated with greater bone density at the beginning of the study, but alcohol consumption did not significantly add to HRT’s beneficial effects on bone density during the study period (Writing Group for the PEPI Trial 1996).

Finally, it is well established that excessive alcohol intake is associated with increased risk of skeletal fractures; many of these fractures probably result from impaired balance and more frequent falls, the most common cause of fractures.

## Breast Cancer

**Figure 3:**
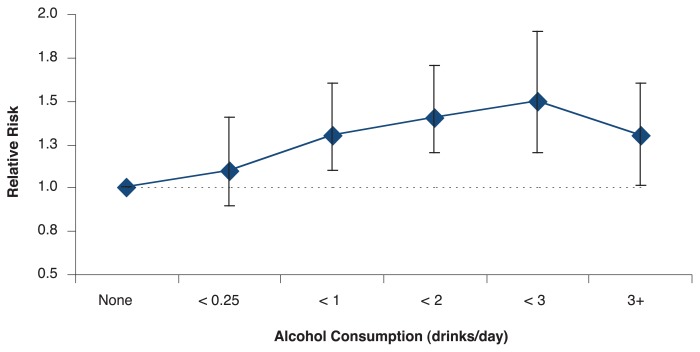
In a study of 242,010 women, of whom 1,442 died of breast cancer by the 14-year followup, alcohol consumption was associated with an increased risk of fatal breast cancer among postmenopausal women but not among premenopausal women or women in transition to menopause (perimenopause). SOURCE: Feigelson et al. (2001).

There is evidence from a number of epidemiological studies that alcohol consumption, perhaps even at moderate levels, is associated with increased breast cancer risk. In the Cancer Prevention Study II, a nationwide prospective study of 1.2 million adults, cross-sectional data on alcohol consumption were obtained from about 250,000 women between the ages of 30 and 104 (Thun et al. 1997). Mortality from breast cancer was found to be significantly higher in women who reported consuming at least one drink per day, compared with nondrinkers. The Pooling Project of Prospective Studies of Diet and Cancer, which included six large studies, estimated that women’s breast cancer risk increased about 9 percent for every 10 grams of alcohol consumed per day, from zero to more than 60 grams (i.e., four to five drinks) per day (Smith-Warner et al. 1998). These studies considered pre- and postmenopausal women together. Feigelson and colleagues (2001) studied 242,010 women, of whom 1,442 had died of breast cancer by the 14-year followup. In this study, alcohol consumption was associated with an increased risk of fatal breast cancer among postmenopausal women (see figure 3) but not among premenopausal women or women in transition to menopause (perimenopause). Other studies have shown that alcohol consumption increased the risk of breast cancer in 23- to 30-year-old premenopausal women (Garland et al. 1999).

How alcohol increases breast cancer risk is poorly understood. In post-menopausal women, the potential increase in breast cancer risk because of alcohol consumption appears to be exacerbated by the use of HRT, which is also a modest risk factor for breast cancer (Nelson et al. 2002). Because drinking impairs the body’s ability to eliminate estrogens, it may augment the breast cancer risk associated with HRT. At the same dose of exogenous estrogen, women who consume one drink per week have been found to have up to three times higher estrogen concentrations than nondrinkers (Ginsberg 1999). Gavaler (2002) showed that current alcohol consumption in women on HRT was associated with a 79-percent increase in the chance of having relatively high blood estrogen concentrations of 45 picograms per milliliter or greater. These findings are consistent with the idea that overall exposure to exogenous estrogen may be greater in drinkers than in nondrinkers.

**Figure 4:**
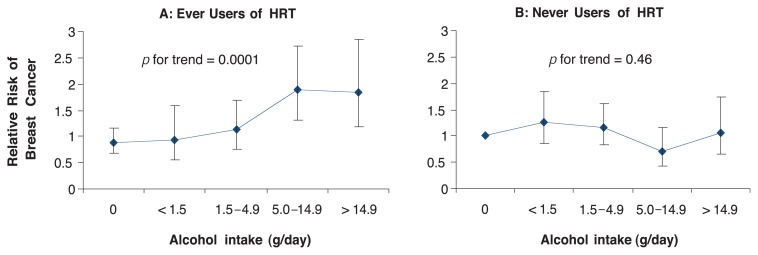
Women who had ever used hormone replacement therapy (HRT) who drank an average of half a drink per day (5.0 g per day) or more had an increased risk of breast cancer relative to nondrinkers (A). In contrast, among women who had never used HRT, there was no difference in breast cancer risk between nondrinkers and moderate drinkers (more than 14.9 g per day) (B). SOURCE: Gapstur et al. (1992).

Extending these investigations of the link between drinking and estrogen levels, other studies have directly examined whether combined alcohol consumption and HRT may contribute to breast cancer risk among postmenopausal women. For example, Gapstur and colleagues (1992) found that women on HRT who drank an average of half a drink per day (5 g per day) or more had an increased risk of breast cancer relative to nondrinkers (see figure 4A). In contrast, among women who had never used HRT there was no difference in breast cancer risk between non-drinkers and moderate drinkers (more than 14.9 g per day; see figure 4B). This finding may have important implications for HRT users who consume alcohol.

In addition to such evidence of estrogen-mediated effects of alcohol on breast cancer, studies of chemically induced breast cancer in rodents suggest that alcohol contributes to the initial cellular damage that leads to cancer formation, possibly by impeding the liver’s ability to metabolize and clear cancer-causing chemicals (i.e., carcinogens) from the body. Specifically, alcohol increases the activity of liver enzymes that may activate carcinogens, and decreases the activity of liver enzymes that process carcinogens for removal. Furthermore, the liver converts alcohol to a toxic chemical (i.e., acetaldehyde). At high levels, this chemical can cause damage to genetic material in breast cells, which may lead to breast cancer. Alcohol may also indirectly contribute to DNA damage by depleting the body of cancer-protective dietary antioxidants and vitamins, such as carotenoids, folate, and vitamin C (Glade 1997).

In contrast to this evidence that alcohol may contribute to the early stages of breast cancer development, there is less evidence for alcohol’s involvement in later stages of breast cancer progression. Although alcohol has been shown to increase the circulating concentrations of hormones that could stimulate breast cancers, clinical observations show only weak evidence that alcohol exposure increases risk of tumor progression once cancer has arisen (Rock and Demark-Wahnefried 2002). Similarly, animal studies do not show an adverse effect of alcohol consumption on tumor progression (Singletary and Gapstur 2001).

## Interim Summary and Further Considerations

The current data suggest that in post-menopausal women, light-to-moderate alcohol consumption may decrease the risk for coronary heart disease and increase bone mineral density; however, it may also increase the risk of breast cancer. This clinical picture fits with the hypothesis that alcohol consumption increases the concentration of estrogen in the blood. However, there are difficulties in interpreting the human data on alcohol’s influence on the occurrence and outcome of these diseases.

### Nonrandom Assignment to Drinking Conditions

As much of the research described above illustrates, whether drinking has an adverse or a beneficial effect on the health of postmenopausal women depends in part on the amount of alcohol they consume (dose dependence) and in part on the type of alcohol consumed (because of antioxidants and other biologically active components in wine and other alcoholic beverages) (for a review, see Chou et al. 1998). In many areas of health, moderate alcohol consumption has a different effect than light or heavy drinking. For other conditions, this dose dependence is less clear. Uncertainties about the health effects of the quantity of alcohol consumed are difficult to resolve because long-term randomized trials of alcohol exposure have not been done in humans. Thus, most of our knowledge of how alcohol affects postmenopausal women’s risk for CHD, osteoporosis, and breast cancer has been derived from observational studies with self-selected populations and self-reported levels of alcohol consumption. Greater use of animal models, in which alcohol exposure can be carefully controlled, is necessary to obtain a better understanding of the mechanisms by which varying levels and types of alcohol consumption affect disease risk.

### Concerns About Control Groups in Alcohol Research

In the human studies discussed above, investigators calculate drinkers’ risk of disease as it relates to the rate of disease in a control group of nondrinkers; hence, the characteristics of this nondrinking group are critical to the interpretation of the data. Studies have consistently found that alcohol consumers differ considerably from nondrinkers in a variety of characteristics besides their alcohol consumption habits. Compared with drinkers, some research indicates that nondrinkers tend to perceive their health as poorer, have worse health, and be of lower socioeconomic status (Fillmore et al. 1998).

In addition, many previous studies have used nondrinking control groups that included both lifetime abstainers and former drinkers. Former drinkers often have stopped drinking because of health problems, are in poorer health, and smoke more than lifetime abstainers do, all of which may increase their mortality rate. For example, in the Nurses’ Health Study (Fuchs et al. 1995), abstainers who were former heavy drinkers or who had stopped drinking because of illness were considered separately and found to have a higher mortality rate than long-term nondrinkers. As these findings illustrate, characteristics other than nondrinkers’ abstention from alcohol may account for their higher mortality (Fillmore et al. 1998). (See table 2 for a more detailed consideration of differences between former drinkers and lifetime abstainers.)

### The Problem of Reliance on Self–Reported Alcohol Consumption

A final difficulty with many human studies of alcohol and health is their dependence on self-reported alcohol consumption. Many studies indicate that people’s reports of their alcohol consumption—assessed immediately and again (by recall) a year or more later—are reliable from one time to the next. However, these studies do not address the *accuracy* of alcohol consumers’ self-reports, which is difficult to examine directly because it would require constant observation of human subjects (see below). The few studies that have investigated the accuracy of self-reports of alcohol consumption suggest that such data tend to be inaccurate. For example, Poikolainen (1985) studied 58 working-class men, ages 22 to 55, in a bar, informing them that they did not have to pay for their drinks for the evening and then observing their actual alcohol consumption. The next day only 22 percent of the men accurately reported how many drinks they had had the night before. Seventy-one percent of the men underestimated their alcohol consumption, and the degree of underestimation increased with increasing alcohol consumption. These findings, although they address only men’s recall of their alcohol consumption, are troubling because they suggest that people in general may be unlikely to provide accurate estimates of their drinking.

Gavaler and Love (1992) studied the relationship between 2 types of self-report in 128 postmenopausal women: questionnaire-based self-report and food diaries. For 35 percent of the sample, these two alcohol consumption estimates did not agree; the pattern of under- and over-reporting was inconsistent. The diary estimates of alcohol consumption correlated more strongly with a biologic measure (serum estradiol levels) that appears to be related to alcohol consumption than did the data obtained by questionnaire.

## Summary

Current research suggests that in post-menopausal women, the beneficial effects of moderate alcohol consumption on CHD risk, and perhaps on bone mineral density, may outweigh the heightened risk for developing breast cancer. Variations in people’s risk factors for heart disease, breast cancer, and osteoporosis affect the risk that alcohol use presents. However, more studies are needed to determine how variations in the amount and type of alcohol (wine, beer, or liquor) affect the relationship between alcohol use and human health, as well as how factors such as endogenous and exogenous hormones interact with alcohol to influence health in post-menopausal women. Likewise, additional work in animals may shed light on potential mechanisms involved in the apparently diverse health effects of moderate alcohol consumption. Finally, epidemiological data on alcohol’s potential health effects in post-menopausal women should be interpreted carefully, given the diversity within nondrinking control groups, inaccuracies of self-report, and lack of studies in which subjects are randomly assigned to drinking conditions.

## Figures and Tables

**Table 1:** Definitions of Light, Moderate* and Heavy Drinking in Women

	Volume	Frequency
**Light**	< 0.25 g/kg	< One drink/day
**Moderate**	0.25–0.50 g/kg	One to two drinks/day
**Heavy**	> 0.50 g/kg	> Two drinks/day

* Definitions of “moderate” alcohol consumption vary widely; one definition relates to levels associated with a low risk of adverse effects (Dufour 1999). The U.S. Department of Health and Human Services and U.S. Department of Agriculture’s definition of moderate alcohol consumption (2000) varies by sex: In women, moderate drinking is considered to be no more than one drink/day, compared with no more than two drinks per day for men. These differences stem from gender differences in body composition and metabolism.

NOTE: The National Institute on Alcohol Abuse and Alcoholism (NIAAA) defines a standard drink as 11–14 g of alcohol. This corresponds to approximately 1.5 ounces of 80-proof distilled spirits (about 14 g alcohol), one 5-ounce glass of wine (11 g), or one 12-ounce beer (12.8 g).

**Table 2:** Nondrinking Control Group Characteristics

Relative to lifetime abstainers, Former drinkers	Are more likely to:
**Both men and women**	Smoke heavily
	Have poor health
	Use marijuana
**Among women only**	Be better educated
	Be unmarried
	Not be religious
**Among men only**	Have less education
	Be unemployed
	Have lower socioeconomic status
	Exhibit more depression

SOURCE: Fillmore et al. 1998 (adapted).

## Footnotes

1 The adverse effects of excessive alcohol consumption are well known and are addressed only briefly in this review.

2 Endogenous steroids are hormones that occur naturally in the body; exogenous steroids, whether natural or synthetic, are administered to the body.

3 However, recent studies suggest little or no benefit when combined estrogen–progestogen therapy is initiated after several years of estrogen deficiency (Writing Group for Women’s Health Initiative Investigators 2002). The effects of estrogen taken without progestogen in this study remain to be determined.

4 The National Institute on Alcohol Abuse and Alcoholism defines a standard drink as 11 to 14 g of alcohol. This corresponds to approximately 1.5 ounces of 80-proof distilled spirits (about 14 g alcohol), one 5-ounce glass of wine (11 g), or one 12-ounce beer (12.8 g).

5 “Relative risk” refers to the statistical likelihood of an event (in this case, death) compared with a control group. A value of 1.20 relative risk represents a 20-percent higher risk than the control group, and a value of 0.8 means a 20-percent lower risk than the control group.

## References

[b50-299-307] Albert C, Manson J, Cook N (1999). Moderate alcohol consumption and the risk of sudden cardiac death among U.S. male physicians. Circulation.

[b51-299-307] Camargo CA, Stampfer MJ, Glynn RJ (1997). Moderate alcohol consumption and risk for angina pectoris or myocardial infarction in U.S. male physicians. Annals of Internal Medicine.

[b52-299-307] Gaziano J, Gaziano T, Glynn R (2000). Light-to-moderate alcohol consumption and mortality in the Physicians’ Health Study enrollment cohort. Journal of the American College of Cardiology.

[b53-299-307] Mukamal KJ, Conigrave KM, Mittleman MA (2003). Roles of drinking pattern and type of alcohol consumed in coronary heart disease in men. New England Journal of Medicine.

